# Editorial: The Role of the Environment in Autoimmunity

**DOI:** 10.3389/fimmu.2021.641171

**Published:** 2021-02-22

**Authors:** James J. Pestka, K. Michael Pollard, Allen J. Rosenspire

**Affiliations:** ^1^Department of Microbiology and Molecular Genetics, Michigan State University, East Lansing, MI, United States; ^2^Institute for Integrative Toxicology, Michigan State University, East Lansing, MI, United States; ^3^Department of Molecular Medicine, Scripps Research, La Jolla, CA, United States; ^4^Department of Biochemistry, Microbiology and Immunology, Wayne State University, Detroit, MI, United States

**Keywords:** environment, trigger, toxicant, autoimmunity, inflammation, autoantibody, lupus (SLE), tolerance

Preclinical and epidemiologic investigations reveal that environmental factors may impact multiple immune system checkpoints within the framework of an individual's unique genetics to potentiate or attenuate onset and/or flaring and drive the progression of autoimmunity. Examples of environmental factors include anthropogenic chemicals, pesticides, respirable particles, drugs, microbes, and diet. Here we present a themed collection containing five reviews, eight original research articles, and one opinion from leading scientists who have contributed to our current understanding of the role of environment on autoimmune disease. This compilation is dedicated to the memory of Dr. Noel Rose who passed away this past year (1, 2). Dr. Rose, universally recognized as the father of autoimmune research, stressed from the beginning the fundamentally intertwined roles of genetics and environment in autoimmune disease. He was a mentor and role model for countless immunologists including the guest associate editors.

A striking feature of environmental factors that affect autoimmunity is their astounding diversity. This Research Topic, although barely scratching the surface, discusses some of these factors currently under investigation. For instance, there is building evidence suggesting that atmospheric particulate matter (PM) exposures aggravate a range of autoimmune diseases. AHR, a ligand activated transcription factor, is activated by organic toxicants including those present in PM such as dioxins and polycyclic aromatic hydrocarbons. The comprehensive review by O'Driscoll and Mezrich focuses on the AHR as a potential mechanistic target for modulating T cell responses associated with PM-mediated autoimmune disease citing the most recent literature on the role of this transcription factor in autoreactive T cell function and autoimmune disease.

The industrial solvent trichloroethylene (TCE), a drinking water pollutant, has been implicated in CD4^+^ T cell-mediated autoimmunity. In mice, these effects were sustained in adult mice after developmental and early life exposure to TCE. Byrum et al. determined whether these persistent effects are associated with epigenetic changes by assessing methylation of CpG sites in autosomal chromosomes in activated effector/memory CD4^+^ T cells The investigators found that developmental exposure to TCE differentially methylated binding regions of polycomb group (PcG) proteins in effector/memory CD4^+^ cells that lasted into adulthood.

Farming and pesticide use have been linked to systemic autoimmune diseases, however, the role of specific pesticides is unclear. In an elegant study, Parks et al. related serum antinuclear autoantibodies (ANAs) in 668 male farmers to lifetime use of 46 different pesticides. ANAs were positively associated with exposure to the fumigant methyl bromide, the carbamate insecticide aldicarb, and combined use of four cyclodiene organochlorine insecticides but inversely associated with non-cyclodiene organochlorine insecticides. Accordingly, specific organochlorine insecticides might pose a risk of developing systemic autoimmunity.

Exposure to respirable crystalline silica in occupations such as construction, mining, and farming is associated with development of autoimmune diseases including systemic lupus erythematosus (lupus). In the lupus-prone female NZBWF1 mouse, weekly repeated airway exposure over 1 month to silica triggers pulmonary ectopic lymphoid neogenesis, systemic autoantibody elevation, and glomerulonephritis. Bates et al. report that within 1 week after silica exposure, increases in mRNAs associated with chemokine release, cytokine production, sustained interferon activity, complement activation, and adhesion molecules were evident in the lung that increased over the course of the 3-month long experiment. Aberrant expression of innate and adaptive immune genes was observed later in the spleen and kidney. These findings suggest that upon silica exposure, the lung functions as the central autoimmune nexus for launching systemic autoimmunity and ultimately, glomerulonephritis. In a related contribution, Foster et al. used normal C57BL/6 and lupus-prone BXSB, MRL, and NZB strains and mice carrying an autoantibody transgene on each of these backgrounds to elucidate how silica lung exposure affects B cell tolerance and unleashes autoreactive B cells. Their findings suggest that silica-induced lung injury has systemic effects that subtly influence autoreactive B cell regulation, perhaps modulating B cell anergy, and that can be revealed by exposure to TLR ligands or other immunostimulants.

Environmental encounters with microbes including viruses and bacteria can influence development of autoimmunity. Notably, exposure to Epstein-Barr virus (EBV) infection is a risk factor for multiple rheumatic diseases. Xuan et al. assessed the association of EBV antibodies with Sjögren's syndrome (SjS) and found that patients with SjS had both a significantly higher prevalence and titers to EBV early antigen and higher titers to EBV viral capsid antigen than case controls. Subsequent meta-analysis of 14 seroepidemiological studies found associations between SjS and antibodies to EBV early antigen and viral capsid antigen.

Antiphospholipid antibodies (aPL), a large family of heterogeneous autoantibodies with the affinity toward negatively charged phospholipids or protein-phospholipid complexes, potentially contribute to antiphopholipid syndrome (APS) leading to vascular thrombosis and miscarriage. The review by Martirosyan et al. focuses on how potential environmental triggers such as viruses, bacteria, fungi, parasite, vaccines, drugs, and other factors induce aPL and might contribute to APS in genetically susceptible individuals.

Gut microbiome dysbiosis has been linked to the onset of different autoimmune diseases. Khan and Wang review recent epidemiological and mechanistic evidence connecting environmental exposures with various autoimmune diseases. They additionally discuss how gut microbiome composition changes might influence disease pathogenesis, particularly in response to exposure to environmental chemicals.

In an Opinion paper, Frew extends the hygiene hypothesis by posing the provocative proposition, based on overlapping gene associations identified by GWAS, that pathogen-specific positive selection might explain disparate rates of autoimmune and allergic disease in different human populations.

Diet selection taken in the context of the underlying genetic background can exacerbate or delay the development of autoimmune diseases. Patients with lupus have increased prevalence of metabolic syndrome but the underlying mechanisms are unknown. Activation of TLR 7 by single stranded-RNA has a critical role in antimicrobial host defense and in the initiation and progression of lupus both in mice and humans. Hanna Kazazian et al. demonstrated that TLR7 signaling plays an essential role high fat diet-induced metabolic syndrome and exacerbation of lupus autoimmunity in TLR8-deficient mice. They further posit that this TLR might be a novel target in tailored therapy in lupus and metabolic diseases. Also related to diet, Song et al. provide a meta-analysis of 22 clinical studies to estimate the association of thyroid autoimmunity and dysfunction with obesity. They found that obesity was significantly correlated with positive thyroid peroxidase antibody and was associated with an increased risk of Hashimoto's thyroiditis, suggesting that prevention of obesity is crucial for thyroid disorders.

Preclinical studies suggest that consumption of ω-3 polyunsaturated fatty acids (PUFAs) found in fish oil might be used as an intervention against the development of autoimmune diseases. Li et al. reviewed clinical studies using ω-3 fatty acids as interventions against lupus, rheumatoid arthritis, type 1 diabetes (T1D) and multiple sclerosis. While many clinical studies found positive effects, some didn't. These discrepancies are likely related to different dose, source, and duration of ω-3 PUFAs treatments. A key limitation of many of these studies was the lack of data on blood concentrations of the ω-3s DHA and EPA of the enrolled patients, making it difficult to evaluate if the enrolled patients for their studies have indeed gained sufficient ω-3 PUFAs in their diet.

The anti-inflammatory and pro-resolving effects of ω-3 PUFAs are inextricably linked to their presence in membrane phospholipids which can be ready measured in red blood cells (RBCs) and reflected as two biomarkers- the ω-3 highly unsaturated fatty acid (HUFA, i.e., PUFA of ≥ C20) score and the Omega-3 Index (O3I). Using data from prior preclinical DHA supplementation studies in the silica-triggered NZBWF1 mouse model, Wierenga et al. found that increases in both the ω-3 HUFA score (>40%) and the O3I (>10%) were strongly associated with suppression of silica-triggered lupus. These ω-3 HUFA scores and O3I thresholds are attainable in humans and could be targeted in future ω-3 PUFA intervention studies for autoimmune diseases. Finally, at the mechanistic level, silica-induced inflammasome activation is believed to be a critical first step for autoimmune triggering. Wierenga et al. found that DHA pre-incubation suppressed silica-induced inflammasome activation and release of IL-1β and IL-1α in macrophages and that these suppressive effects were linked to inhibition of LPS-induced *Nlrp3, Il1b*, and *Il1a* transcription, potentially through the activation of the transcription factor PPARγ.

In conclusion, this collection provides new perspectives on how selected environmental influences impact autoimmunity, particularly in genetically susceptible individuals. We hope that this topic will stimulate further advances in the field that can be translated into preventive and intervention strategies to counter autoimmune disease.

## Author Contributions

JP, KP, and AR contributed to writing and editing this editorial. All authors contributed to the article and approved the submitted version.

## Conflict of Interest

The authors declare that the research was conducted in the absence of any commercial or financial relationships that could be construed as a potential conflict of interest.

## References

[1] Watts G. Noel Richard Rose. Lancet. (2020) 396:880. 10.1016/S0140-6736(20)31975-933433328

[2] Scott DW Caspi RR Moudgil KD Noel R. Rose 1927–2020. Nat Immunol. (2020) 21:1306. 10.1038/s41590-020-0790-6

